# A new statistical method for curve group analysis of longitudinal gene expression data illustrated for breast cancer in the NOWAC postgenome cohort as a proof of principle

**DOI:** 10.1186/s12874-016-0129-z

**Published:** 2016-03-05

**Authors:** Eiliv Lund, Lars Holden, Hege Bøvelstad, Sandra Plancade, Nicolle Mode, Clara-Cecilie Günther, Gregory Nuel, Jean-Christophe Thalabard, Marit Holden

**Affiliations:** Department of Community Medicine, Pb. 5060, UiT The Arctic University of Norway, 9037 Tromsø, Norway; Norsk Regnesentral, Oslo, Norway; INRA, UR1404 Unité Mathématiques et Informatique Appliquées du Génome à l’Environnement, Jouy-en-Josas, France; CNRS, INSMI Stochastics and Biology Group (PSB) LPMA, UPMC, Sorbonne University, Paris, France; MAP 5, Université Paris Descartes, Sorbonne Paris Cité, Paris, France

**Keywords:** Transcriptomics, Gene expression, NOWAC postgenome cohort, Breast cancer, Carcinogenesis, Metastasis, Mammographic screening, Blood, Systems epidemiology

## Abstract

**Background:**

The understanding of changes in temporal processes related to human carcinogenesis is limited. One approach for prospective functional genomic studies is to compile trajectories of differential expression of genes, based on measurements from many case-control pairs. We propose a new statistical method that does not assume any parametric shape for the gene trajectories.

**Methods:**

The trajectory of a gene is defined as the curve representing the changes in gene expression levels in the blood as a function of time to cancer diagnosis. In a nested case–control design it consists of differences in gene expression levels between cases and controls. Genes can be grouped into curve groups, each curve group corresponding to genes with a similar development over time. The proposed new statistical approach is based on a set of hypothesis testing that can determine whether or not there is development in gene expression levels over time, and whether this development varies among different strata. Curve group analysis may reveal significant differences in gene expression levels over time among the different strata considered. This new method was applied as a “proof of concept” to breast cancer in the Norwegian Women and Cancer (NOWAC) postgenome cohort, using blood samples collected prospectively that were specifically preserved for transcriptomic analyses (PAX tube). Cohort members diagnosed with invasive breast cancer through 2009 were identified through linkage to the Cancer Registry of Norway, and for each case a random control from the postgenome cohort was also selected, matched by birth year and time of blood sampling, to create a case-control pair. After exclusions, 441 case-control pairs were available for analyses, in which we considered strata of lymph node status at time of diagnosis and time of diagnosis with respect to breast cancer screening visits.

**Results:**

The development of gene expression levels in the NOWAC postgenome cohort varied in the last years before breast cancer diagnosis, and this development differed by lymph node status and participation in the Norwegian Breast Cancer Screening Program. The differences among the investigated strata appeared larger in the year before breast cancer diagnosis compared to earlier years.

**Conclusions:**

This approach shows good properties in term of statistical power and type 1 error under minimal assumptions. When applied to a real data set it was able to discriminate between groups of genes with non-linear similar patterns before diagnosis.

## Background

The assumption of systems epidemiology [1] is that functional aspects of the human carcinogenic process can be detected in the blood as gene expression patterns before cancer diagnosis, either as active signals or as passive information. A recent editorial in *Nature Medicine* [2] stressed that if we are to understand the carcinogenic process, research needs to shift from mouse models to a “human model”. However, the peculiarities and time scale of cancer development in humans impose to rely essential on observational studies, The prospective design is clearly the best design if one wants to incorporate the time aspect of carcinogenesis and changing exposures. However, practical considerations frequently force us to use a nested case-control design within the cohort, which keeps part of the advantage of the previous design. Analyses of somatic mutations in cancer genome studies have revealed the huge diversity of mutational processes that occurs during carcinogenesis [3]. One explanation for this observation could be that multiple mutational processes operate differently within biological processes depending on subtypes of cancer, thus giving a jumbled composite signature. In order to avoid jumbled composite signatures, functional analyses in observational studies must be stratified by important clinical information like lymph node status and exposures to potential carcinogens.

One approach for prospective functional genomic studies is to compile trajectories based on measurements from many case-control pairs in order to study the carcinogenic process [4]. The trajectory of a gene is defined as the curve showing the changes in gene expression levels in the blood as a function of time to cancer diagnosis, and consists in a nested case-control design of the differences in gene expression levels between cases and controls.

Our overall aim was to develop statistical methods for exploring the changes in gene expression in years before diagnosis as part of a processual approach [5], not to estimate risk.

There is no prior knowledge about the form of the trajectory of gene expression for any of the thousands of genes. This lack of a priori information normally demands an agnostic approach [6], i.e., considering all genes as equal and adjusting for multiple testing using a false discovery rate [7]. However, here we present a new statistical method to study trajectories. We applied this new method in a prospective analysis of women with breast cancer in the Norwegian Women and Cancer (NOWAC) postgenome cohort [8]. The trajectories were analyzed within strata of different biological stages in carcinogenesis of breast cancer within the screening or outside as clinical cancer, but without identifying single genes or conducting pathway analyses.

## Methods

The new statistical approach are described below. As a “proof of concept” we carried through an analysis in a nested case-control design in the Norwegian Women and Cancer postgenome cohort. For each incident breast cancer case identified through linkage to the Norwegian Cancer Registry a control was drawn from blood samples collected at the same time and year of birth. This ensured the same storage time and no effect of age between cases and controls. The pairs of cases and controls were kept together throughout all laboratory procedures in order to reduce batch effects. For more details see later under Epidemiological design and study population.

### Statistical methods

**Fig. 1:**
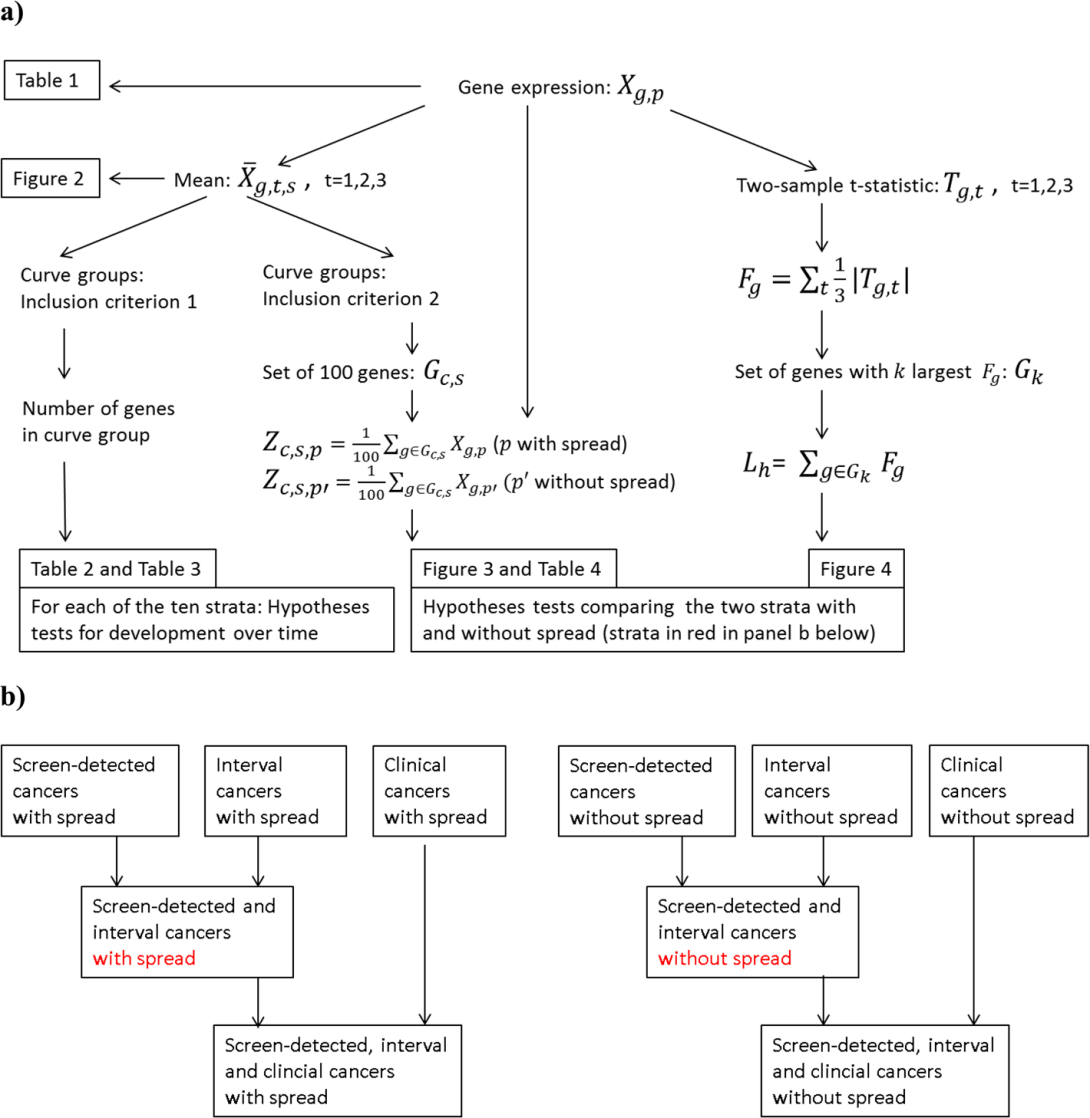
Overview of hypothesis tests, variables, strata, tables and figures. **a** Illustration of the relationship between the data *X*_*g*,*p*_, the different hypothesis tests, the variables used in these tests, and which tables and figures that show the results from the tests. **b** Overview of the different strata

The new statistical method for curve group analyses is a statistical method based on a set of hypothesis testing that we developed in order to detect changes in gene expression levels over time, and whether these changes, if they exist, differ among strata. This method is able to identify small changes that vary slowly over time and/or among strata, by using a large number of genes in each analysis. In order to define test statistics that measure the development of differential gene expression levels over time and differences among strata, we have introduced the concept of curve groups, where each curve group consists of genes that have a similar development over time, i.e., similar differential trajectories. These methods are described in detail below:

Let *X*_*g*,*p*_ be the log_2_-expression difference for gene *g* and the matched case-control pair *p*. Each case-control pair belongs to a stratum *s* and a time period *t*. We wanted to test whether *X*_*g*,*p*_ is independent of the time period, and whether there is no difference among the strata, i.e., *X*_*g*,*p*_ is independent of stratum. Figure 1 gives an overview of the different tests and the variables used in these tests, the strata used in the analyses and the table and figures where the results are shown.

In the illustrative application, analyses were either conducted within strata of lymph node status at breast cancer diagnosis (positive or ‘with spread’ and negative or ‘without spread’) or with respect to breast cancer screening visits (detection categories); cancers diagnosed during screening visits were considered ‘screen-detected cancers’; cancers diagnosed within 2 years of last screening visit were considered ‘interval cancers’; and cancers diagnosed clinically in women that did not attend screening or had not attended screening for more than 2 years were considered ‘clinical cancers’ (Table 1).

**Table 1:** Number of case-control pairs in each stratum and time period with gene expression data *X*_*g*,*p*_

Strata		Year before diagnosis (time period)		
Detection category	Lymph node status	5-3 (3)	2 (2)	1 (1)
Screen-detected cancers^a^	With spread	41	11	6
	Without spread	118	42	43
Interval cancers^b^	With spread	28	9	6
	Without spread	30	15	10
Clinical cancers^c^	With spread	11	8	10
	Without spread	28	12	13

^a^Diagnosed at a screening visit

^b^Diagnosed within 2 years of a screening visit

^c^Diagnosed clinically and did not attend the screening program or diagnosed clinically more than 2 years after a screening visit

### Hypothesis tests for development over time in each stratum

For each stratum we tested whether *X*_*g*,*p*_ is independent of the time period in a global test since we are interested in weak signals from many genes, not signal that may only be identified in a single gene. To define a test statistic that measures development over time we used curve groups. The follow-up time was divided into three time periods *t* = 1, 2, 3 where t = 1 is 0-1 year before cancer diagnosis, t = 2 is 1-2 years before cancer diagnosis, and t = 3 is 3-5 years before cancer diagnosis.

**Fig. 2:**
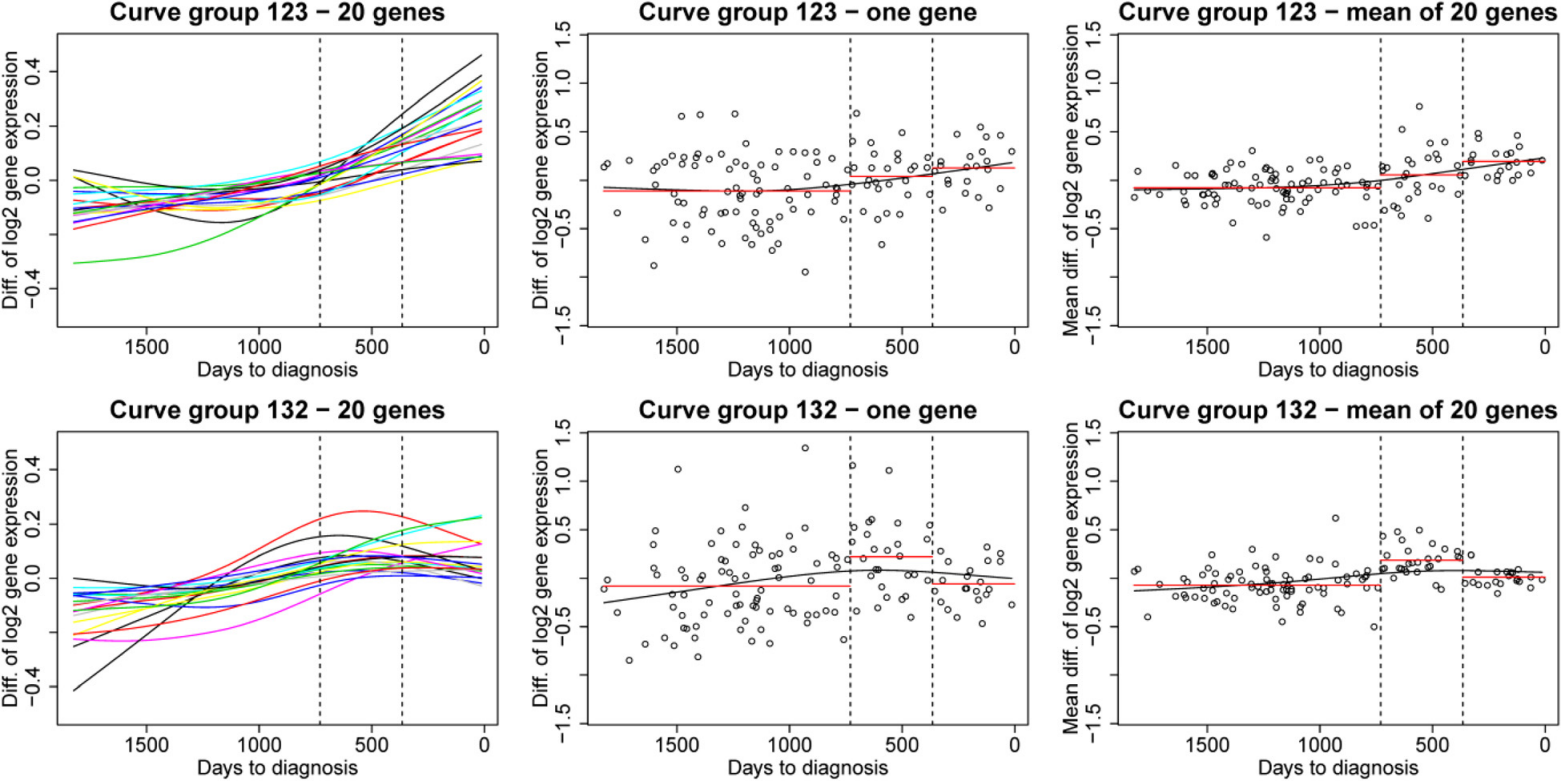
Examples of curve groups according to time to diagnosis. Example of two different curve groups: curve group ‘123’ (*upper panel*, gene expression values increasing with time) and curve group‘132’ (*lower panel*, highest gene expression value in the middle time period). In the left panels curves with the gene expression differences *X*_*g*,*p*_ for 20 genes from the given curve group are plotted. For illustrational purposes, the curves have been estimated from the data using splines. In the middle panels the data *X*_*g*,*p*_ for one of the 20 genes are shown with the corresponding spline-estimated curve. The points represent the differences in gene expression *X*_*g*,*p*_ for each case-control pair. The mean value in each time period, ${\overline{X}}_{g,3,s}$, ${\overline{X}}_{g,2,s}$ and ${\overline{X}}_{g,1,s}$, is shown in red. The right panels are similar to the middle panels except that the data points that are plotted are the mean values computed over the 20 genes in the left panel
- Each curve group included only genes with a significant change in expression level over time. This was done by testing whether the smallest and largest values of ${\overset{-}{X}}_{g,3,s}$, ${\overset{-}{X}}_{g,2,s}$ and ${\overset{-}{X}}_{g,1,s}$ were different using a two-sample t-test (assuming unequal variances). Let *p*_*g*,*c*_ be the p-value of this test. Depending on the statistical question at hand, we defined two alternative criteria for concluding that a gene *g* belongs to the curve group *c*:

- For a given stratum *s*, a gene *g* can belong to zero or one of six curve groups based on the average (mean) of the data over all case-control pairs in the stratum in each of the three time periods. These averages were denoted ${\overset{-}{X}}_{g,3,s}$, ${\overset{-}{X}}_{g,2,s}$ and ${\overset{-}{X}}_{g,1,s}$, respectively, and the curve groups are defined based on the ordering of these three averages. In order to search for curves with changes over time, we defined six potential curve groups that changed from time period to time period, called «123, 132, 213, 231, 312, and 321», respectively. The three numbers that denote each curve group represent the level of the average gene expression of time period 3 (left number), the level of the average gene expression of time period 2 (middle number) and the level of the average gene expression of time period 1 (right number). For example, if ${\overset{-}{X}}_{g,3,s}<{\overset{-}{X}}_{g,2,s}<{\overset{-}{X}}_{g,1,s}$, and these three averages are not too similar (to be defined later), gene *g* belong to curve group ‘123’ indicating an increasing gene expression level over time when approaching the time of diagnosis, with gene expression level 1 in time period 3, gene expression level 2 in time period 2 and gene expression level 3 in time period 1 (closest to the time of diagnosis). If the three averages are too similar, gene g does not belong to any curve group. See Fig. 2 for an illustration of the concept of curve groups.

- *Inclusion criterion 1*: Gene *g* belongs to curve group *c* if *p*_*g*,*c*_ is below a predefined limit *α*.
- *Inclusion criterion 2*: Gene *g* belongs to curve group *c* if gene *g* is among the M genes with lowest *p*_*g*,*c*_. See more in the next section.

To test for the development of gene expression levels over time, for each stratum we counted the number of genes that belong to the curve group using inclusion criterion 1. We then performed seven hypothesis tests: one global test and one for each of the six curve groups in each stratum. In the global test the test statistic is the total number of genes that belong to any one of the six curve groups, while in the test for individual curve groups the test statistic is the number of genes that belong to the curve group in question. If the conclusion of the hypothesis test was that there were more genes in the curve groups than what was expected by chance, we concluded that there was a significant development over time for some of these genes.

### Hypothesis test for comparing two strata

Let us consider in our illustrative example ytwo strata like for instance “with spread” and “without spread” at the time of diagnosis. We wanted to test whether there were differences in gene expression levels between these two strata, using information from several genes. For each curve group *c*, stratum *s* and case-control pair *p*, we defined a curve group variable *Z*_*c*,*s*,*p*_ as follows: we selected the genes that belonged to curve group *c* for stratum *s* using inclusion criterion 2 with M = 100. Let *G*_*c*,*s*_ denote this set of genes. The curve group variable *Z*_*c*,*s*,*p*_ for case-control pair *p* was then computed as the average value of the data *X*_*g*,*p*_ over the genes in *G*_*c*,*s*_:

$$ {Z}_{c,s,p}=\frac{1}{100}{\displaystyle \sum_{g\in {G}_{c,s}}}{X}_{g,p}. $$

We could then test whether the variables *Z*_*c*,*s*,*p*_ were different between the two strata for case-control pairs *p* either for all time periods combined or for each time period separately. Note that the genes were selected based on data from stratum *s*, but the variable may have been calculated for case-control pairs *p* in any stratum. For example, assume that we wanted to test if there was a difference in gene expression level between case-control pairs in the stratum with spread versus the stratum without spread for curve group 123. Assume that the set of 100 genes G_123,with spread_ was selected using criterion 2 in the stratum with spread. We would then have calculated Z_123,with spread,p_ for all case-control pairs *p* in the stratum with spread and Z_123,with spread,p_’ for p’ in the stratum without spread, and tested if the difference was larger than expected by chance. Note that testing the strata with spread versus without spread may also be performed with the set of genes G_123,without spread_ selected from the without spread stratum or from any of the other defined strata.

### An alternative statistic for comparing two strata

The test described above focuses on genes that belong to the same curve group. We also constructed a hypothesis test to compare the difference in development over time between two strata that did not depend on curve groups. This test statistic was constructed by first computing the two-sample t-statistic *T*_*g*,*t*_ and comparing the difference in gene expression levels between the two strata for each gene *g* and time period *t*. We defined ${F}_g={\sum}_t{w}_t\left|{T}_{g,t}\right|$ as the weighted sum of the absolute values of the t-statistics for gene *g* with weight *w*_*t*_. Furthermore, the test statistic was defined as ${L}_k={\displaystyle {\sum}_{g\in {G}_k}{F}_g}$, where *G*_*k*_ is the set of genes with the *k* largest *F*_*g*_ values, i.e. *L*_*k*_ is the sum of the *k* largest *F*_*g*_ values. We observe that *L*_*k*_ is a weighted sum of t-statistics. We used equal weights *w*_*t*_ = 1/3 for each time period. Alternatively, the weights could be selected either as proportional to the number of case-control pairs in each time period or with larger values for the case-control pairs in a time period closer to the time of diagnosis. We then performed a global test including all three time periods, and separate tests for each time period, in which only data corresponding to each time period were included. This test performed very well on several simulated datasets with a different development over time or different gene expression levels for some genes for two strata. For details see Holden [9].

### Computing p-values – permutation tests

We computed p-values in all the tests described above by estimating the null distribution for the statistic of the hypothesis test by randomizing the data. In the randomization, we preserve critical properties of the genes (level of expression, complex correlation between genes, etc.) and randomize only what’s connected to the evolution over time and stratum. This randomization defines the null-distribution for the test statistic that is used when finding the p-value. In hypothesis tests for development over time in a single stratum, the null model was estimated by randomizing case-control pairs for that stratum between time periods, while in the hypothesis tests comparing two strata, the null model was estimated by randomizing case-control pairs between the two strata for each time period. Note that these randomization algorithms maintained the correlation structure between the genes for each case-control pair. Also note that the curve groups were redefined before a sample of the null model was computed from a randomized dataset. The p-value of the test was set to $\frac{K+1}{N+1}$, where *N* is the total number of randomizations and *K* is the number of randomizations out of N with a more extreme statistic than the statistic for the real data [10]. In the results presented we used *N* = 1000.

### Illustrative example: epidemiological design and study population

The NOWAC study is a nation-wide population-based cancer study that was initiated in 1991 [11], and the postgenome cohort has been described previously in detail [8]. Briefly, random samples of women were drawn from the Central Person Register by Statistics Norway based on their unique national birth number. Selected women were sent an invitation that included information on blood sample collection and an 8-page questionnaire, on which their national birth number was replaced by a serial number. The linkage file for the national birth number and the serial number was kept at Statistics Norway. The questionnaires were returned to the Department of Community Medicine, University of Tromsø. Non-responders were mailed one or two reminders. Of all invited women, 97.2 % agreed to give a blood sample. These women were sent a blood sampling kit including another 2-page questionnaire and one PAXgene tube (PreAnalytiX GmbH, Hembrechtikon, Switzerland) with a buffer or stabilization agent for mRNA in order to improve the quality of gene expression for genome-wide microarray analyses. These kits were mailed in batches of 500, with one reminder sent after 4–6 weeks. Blood was primarily drawn at family doctors’ offices and the doctors then sent the samples as biological material overnight to Tromsø, where they were immediately frozen. Between 2003 and 2006, 48 692 blood samples were included in the NOWAC postgenome biobank, and these women make up the NOWAC postgenome cohort.

A nested case-control design was chosen in order to reduce batch effects in the laboratory and also for the high cost of each analysis. For each case of breast cancer, a control from the same batch of 500 women in the postgenome cohort was assigned, matched by time of blood sampling and year of birth, to be analyzed together with the case.

The controls are used to establish the average (mean) gene expression level in individuals without cancer and to allow exposure-adjusted analyses to be performed. The expression level of a gene not involved in the carcinogenetic process will exhibit variability dependent on day-to-day changes in exposures such as environment and nutrition, resulting in random fluctuations of the difference in gene expression between case and matched control around a population-average constant over time. Whereas, the difference in expression level of genes related to different stages of the carcinogenetic process may vary over time in a non-random way, thus exhibiting some non-random trend. The changes in genes related to the carcinogenic process could be complicated by other effects of exposures to the carcinogens [4].

### Follow-up and registry information

Cases of invasive breast cancer diagnosed in the NOWAC postgenome cohort through the end of 2009 were identified through linkage to the Cancer Registry of Norway. Altogether 637 cases of invasive breast cancer were reported. After removing outliers and ineligible cases including women with distant metastases, the study consisted of 441 case-control pairs. Information on lymph node status at breast cancer diagnosis was based on the pTNM information included in the Cancer Registry of Norway. Detection categories were also obtained from the Cancer Registry of Norway, which updates this data regularly through linkage to the screening database kept by the National Breast Cancer Screening Program [12].

### Ethical issues

The NOWAC study was approved by the Norwegian Data Inspectorate and the Regional Ethical Committee of North Norway (REK). The linkages of the NOWAC database to national registries such as the Cancer Registry of Norway and registries on death and emigration was approved by the Directorate of Health. The women were informed about these linkages. Furthermore, the collection and storing of human biological material was approved by the REK in accordance with the Norwegian Biobank Act. Women were informed in the letter of introduction that the blood samples would be used for gene expression analyses.

### Laboratory procedures

#### Microarray data

All extraction and microarray services were provided by the Genomics Core Facility, Norwegian University of Science and Technology, Trondheim, Norway. To control for technical variability such as different batches of reagents and kits, day-to-day variations, microarray production batches, and effects related to different laboratory operators, each case-control pair was kept together throughout all extraction, amplification, and hybridization procedures. RNA extraction was performed using the PAXgene Blood miRNA Isolation kit according to the manufacturer’s instructions. RNA quality and purity was assessed using the NanoDrop ND 8000 spectrophotometer (ThermoFisher Scientific; Wilmington, Delaware, USA) and Agilent bioanalyzer (Agilent Technologies, Palo Alto, CA, USA), respectively. RNA amplification was performed on 96 plates using 300 ng of total RNA and the Illumina TotalPrep-96 RNA Amplification Kit (Ambio, Inc., Austin, Texas, USA). The amplification procedure consisted of reverse transcription with a T7 promotor and ArrayScript, followed by a second-strand synthesis. In vitro transcription with T7 RNA polymerase using a biotin-NTP mix produced biotinylated cRNA copies of each mRNA in the sample. All case-control pairs were run on either the IlluminaHumanAWG-6 version three expression bead or the HumanHT-12 version 4. Outliers were excluded after visual examination of dendrograms, principal component analysis plots and density plots. Individuals that were considered borderline outliers were excluded if their laboratory quality measures where below given thresholds (RIN value <7, 260/280 ratio <2, 260/230 ratio <1.7, and 50 < RNA < 500).

#### Preprocessing of microarray data

The dataset was preprocessed as previously described [13]. The dataset, which consisted of 441 case-control pairs and 30 046 probes, was background corrected using negative control probes and normalized on the original scale using quantile normalization. Data from the two Illumina chips (HumanWG-6 v3 and HumanHT-12 v4) were combined on identical nucleotide universal identifiers [14]. We retained probes present in at least 1 % of the individuals, i.e., in at least nine of the 882 individuals. If a gene was represented with more than one probe only one was selected, resulting in a dataset with 11 431 probes. The probes were translated to genes using the IlluminaHumanAll.db database [15]. Finally, the log_2_-differences of the gene expression levels for each case-control pair were computed and used in the statistical analyses. Additional adjustments for possible batch effects were unnecessary as the case-control pairs were kept together throughout the laboratory processes.

## Results

### Hypothesis tests for development over time in each stratum

A time trend was considered to be present if there were more genes in the curve groups than expected by chance. The number of case-control pairs stratified according to lymph node status and detection category is shown in Table 1. First, we stratified all case-control pairs by lymph node status (Tables 2 and 3). The results were not significant, indicating no changes in gene expression levels over time. We then stratified all screening and interval cancers by lymph node status, which rendered a highly significant global test (*p* = 0.01), and more p-values less than 0.05 than expected by chance (Tables 2 and 3). Finally, we stratified by all detection categories and lymph node status. This analysis showed that the effect was mainly restricted to interval cancers with spread (global test; *p* = 0.02) (Tables 2 and 3). In these tests the inclusion criterion 1 had value *α* = 0.01. The results depend on the *α* ‐ value, but the results were not very sensitive to the choice of *α* ‐ value -value (data not shown). Tables 4 and 5 shows the observed number and the expected number of genes in each curve group analysis in Tables 2 and 3. Here it is important to note that the number of genes in each curve group is not too small (Tables 4 and 5). If this had been the case, it would have indicated that the chosen *α* ‐ value -value was too small, weakening the power of the test.

**Table 2:** *P*-values obtained when testing whether there are more genes in the curve groups than what is expected by chance in different strata

*p*-value				
Curve group	Screen-detected, interval, and clinical cancers with spread	Screen-detected, interval, and clinical cancers without spread	Screen-detected and interval cancers with spread	Screen-detected and interval cancers without spread
Global	0.78	0.27	0.01*	0.20
123	0.61	0.23	0.02*	0.39
132	0.49	0.13	0.008*	0.11
312	0.88	0.18	0.13	0.11
321	0.41	0.74	0.02*	0.66
231	0.74	0.68	0.50	0.57
213	0.58	0.17	0.48	0.13

**Table 3:** *P*-values obtained when testing whether there are more genes in the curve groups than what is expected by chance in different strata

*p*-value						
Curve group	Screen-detected cancers with spread	Screen-detected cancers without spread	Interval cancers with spread	Interval cancers without spread	Clinical cancers with spread	Clinical cancers without spread
Global	0.36	0.43	0.02*	0.46	0.40	0.81
123	0.10	0.33	0.21	0.89	0.06	0.34
132	0.38	0.19	0.009*	0.32	0.51	0.63
312	0.83	0.30	0.07	0.21	0.98	0.81
321	0.18	0.90	0.05*	0.40	0.22	0.66
231	0.33	0.63	0.21	0.83	0.94	0.93
213	0.70	0.27	0.29	0.16	0.90	0.59

Inclusion criterion 1 was used with *α* = 0.01. *P-values below 0.05

**Table 4:** Observed number of genes in each curve group and stratum, with expected number of genes in parenthesis

Observed number of genes (expected number of genes)				
Curve group	Screen-detected, interval, and clinical cancers with spread	Screen-detected, interval, and clinical cancers without spread	Screen-detected and interval cancers with spread	Screen-detected and interval cancers without spread
Global	305 (513)	609 (535)	1360 (482)*	708 (547)
123	47 (76)	97 (82)	259 (70)*	69 (86)
132	69 (100)	171 (103)	518 (99)*	205 (107)
312	37 (102)	145 (105)	171 (105)	203 (108)
321	66 (82)	40 (82)	314 (77)*	46 (82)
231	38 (77)	44 (81)	48 (66)	51 (82)
213	48 (76)	112 (82)	50 (65)	134 (83)

**Table 5:** Observed number of genes in each curve group and stratum, with expected number of genes in parenthesis

Observed number of genes (expected number of genes)						
Curve group	Screen-detected cancers with spread	Screen-detected cancers without spread	Interval cancers with spread	Interval cancers without spread	Clinical cancers with spread	Clinical cancers without spread
Global	475 (464)	490 (547)	1233 (485)*	471 (525)	448 (491)	302 (502)
123	139 (75)	78 (85)	101 (81)	33 (90)	233 (84)	83 (83)
132	81 (91)	141 (106)	515 (92)*	96 (97)	52 (84)	54 (90)
312	43 (96)	107 (109)	237 (89)	123 (96)	18 (82)	40 (92)
321	115 (82)	29 (82)	213 (81)*	71 (83)	101 (83)	45 (77)
231	63 (63)	46 (82)	92 (70)	31 (78)	21 (77)	27 (77)
213	34 (58)	89 (83)	75 (73)	117 (81)	23 (81)	53 (83)

*Cases with a p-value below 0.05 from Table 2 and 3

### Hypothesis tests for comparing two strata

**Fig. 3:**
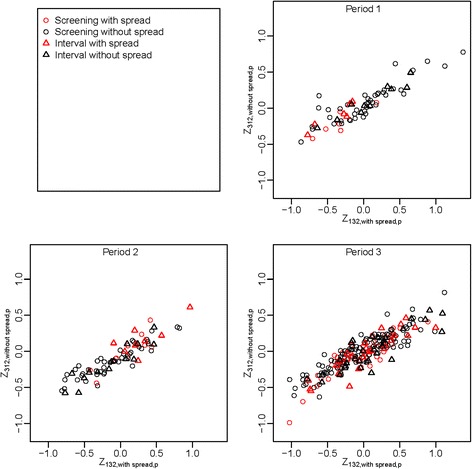
Distribution of case-control pairs for two curve groups stratified on spread in each time period. Plot of two of the most significant curve group variables, *Z*_132,*with spread*,*p*_ and *Z*_312,*without spread*,*p*_, for the three time periods. These variables are the sum of gene expression differences *X*_*g*,*p*_ for genes selected from curve group 132 (high values in middle period) based on data with spread and curve group 312 (low values in middle period) based on data without spread. The data with spread (without spread) are first used to select two sets of genes, one set for each of the two curve-group variables. We may calculate both *Z*_132,*with spread*,*p*_ and *Z*_312,*without spread*,*p*_ for all case-control pairs from all strata. Note that the difference between the two strata varies between the periods

Based on the results from each stratum, we restricted our analysis to compare gene expression levels in the strata «screening or interval with spread» and «screening or interval without spread» using the curve group variable *Z*_*c*,*s*,*p*_ described in the methods section. P-values were obtained by testing whether the curve group variables *Z*_*c*,*s*,*p*_ were different in the two strata; many were below 0.05 and some were smaller than 0.01 (Table 6). In Fig. 3, we illustrated how to use the gene expression data to separate these two strata by showing the curve group variable *Z*_*c*,*s*,*p*_ for each case-control pair *p* in the different strata. The plot shows that the difference between the two strata changes over time for the two most significant *Z*_*c*,*s*,*p*_ variables. The differences between the strata with spread and without spread were larger in the year before diagnosis compared to earlier years, but even these differences were comparatively small.

**Table 6:** *P*-values obtained when testing whether the curve group variables *Z*_*c*,*s*,*p*_ are different for the strata «screen-detected and interval cancers with spread» and «screen-detected and interval cancers without spread»

*p*-value						
	Genes selected based on stratum *s*_1_ = «Screen-detected and interval cancers with spread» ${Z}_{c,{s}_1,p}$			Genes selected based on stratum *s*_2_ = «Screen-detected and interval cancers without spread» ${Z}_{c,{s}_2,p}$		
Time period *t*	3	2	1	3	2	1
N1	69	20	12	69	20	12
N2	148	57	53	148	57	53
Curve group *c*						
123	0.22	0.59	0.02*	0.53	0.11	0.08
132	0.90	0.005*	0.004*	0.71	0.11	0.009*
312	0.80	0.27	0.15	0.04*	0.009*	0.001*
321	0.12	0.98	0.24	0.35	0.72	0.15
231	0.26	0.45	0.78	0.34	0.38	0.23
213	0.53	0.45	0.65	0.36	0.04*	0.08

*P-values below 0.05. ‘N1’ is the number of case-control pairs in the stratum «Screening or interval with spread» in the time period *t*, while ‘N2’ is the number of case-control pairs in the stratum «Screening or interval without spread» in the time period *t*

**Fig. 4:**
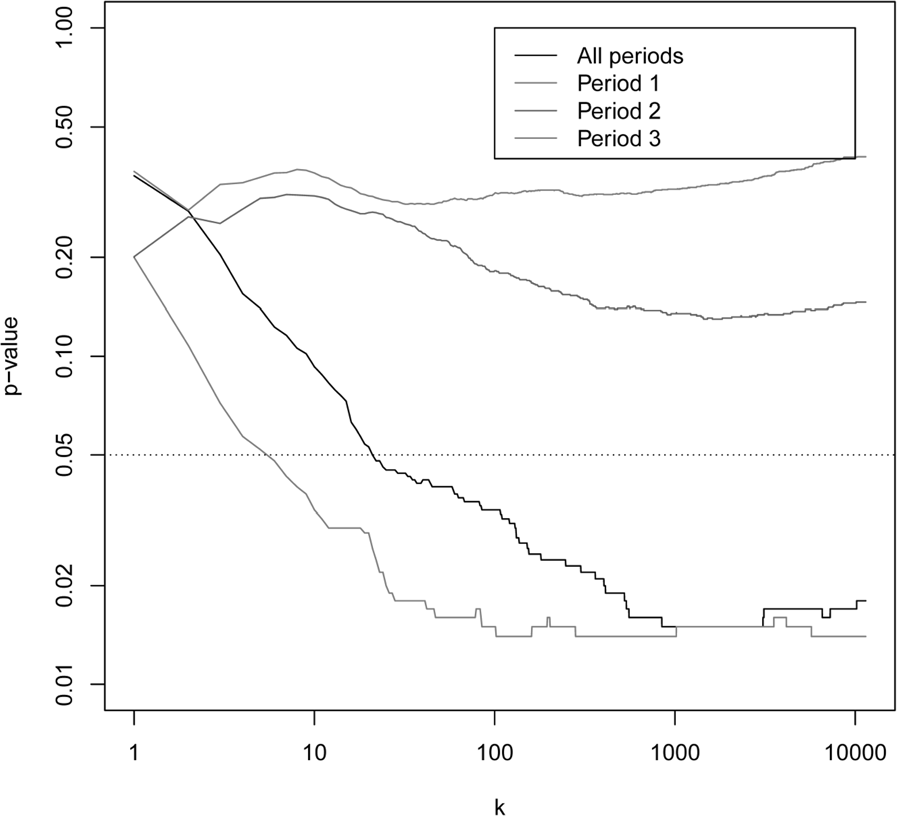
The relationship of p-values to number of genes in the test statistic *L*_*k*_. The p-value in a hypothesis test with test statistic *L*_*k*_, a weighted sum of t-statistics, plotted against the number of genes *k* used in the calculation of *L*_*k*_. The two strata that are compared in the t-statistics that are used for computing *L*_*k*_ are «Screening or interval with spread» and «Screening or interval without spread»

In the methods section we introduced the statistic *L*_*k*_, a weighted sum of t-statistics, as an alternative to the curve group variables *Z*_*c*,*s*,*p*_ for comparing the gene expression levels of two strata. In Fig. 4 we plot the p-value in a hypothesis test with *L*_*k*_ as test statistic against the number of genes *k*. The plot shows that the gene expression levels are different in the two strata. *L*_*k*_ is the sum of the k-largest weighted sums of t-statistics. Note (in Fig. 4) that when we add more and more terms in the sum, the observation becomes more significant. When we used 50 genes, the p-value was about 0.05, and the p-value decreased to below 0.02 when we used the 1000 most significant genes. This indicates that the difference between the strata is present in a large number of genes, but so weak that the strongest result was only obtained when including a large number of genes. Also, time period 1, i.e., 0-1 year before diagnosis, contributed the most to the low p-values, which is in accordance with the results shown in Fig. 3 and Table 6.

## Discussion

This methodological analysis has shown that it is possible to significantly discriminate the time trend of gene expression patterns observed before breast cancer diagnosis. The findings are based on an original approach for the statistical analysis of time-dependent curves of gene expression levels in the NOWAC postgenome cohort. These methods could also be used for other aspects of functional genomics like methylation.

From a statistical point of view, since the publication of the seminal work by Cox [16], the Cox proportional hazard model and its extension have been largely used by epidemiologists to analyze cohort studies with time-dependent covariates. This model has also been adapted to case-control designs [17], and some extensions have been proposed for covariates measured with noise [18] and time-dependent coefficients [19]. More recently, the adjunction of numerous covariates like gene expression data have added some challenging statistical issues [20]. While the characteristics and the basic assumptions of the Cox model have been adapted to the dimensionality and the very specific paired design of the NOWAC postgenome cohort, the Cox model cannot be fully adapted to the estimation of changes in gene expression curves or to the biological interpretations of gene pathways.

The curve group approach can be viewed as an effective method for dimension reduction in studies of functional genomics. The grouping of the curves is not dependent on the individual testing of the curves for the more than 10 000 expressed genes, thus it mostly eliminates the false discovery rate of multiple testing. The strength of the curve group approach can be seen in the statistical power that was achieved even in strata with a low number of cases, such as the six cases with spread in two strata. We stratified the data based on the detection category and lymph node status. The Norwegian Breast Cancer Screening Program uses mammographic screening and started in 1996, with coverage of the entire population starting in 2005 [12]. It has been estimated that the introduction of population-based breast cancer screening in Norway gave a mean sojourn time for invasive cancer of 4.0 years in women aged 50-59 years and 6.6 years for those 60-69 years [21]. Analyses of breast carcinogenesis as a time-dependent process should therefore take into consideration that cases diagnosed within the screening program are diagnosed at an earlier phase of carcinogenesis and thus are not directly comparable to clinically-detected cases. Lymph node status has been the most important prognostic factor for breast cancer survival for 100 years [22, 23]. At time of diagnosis, we had a censored distribution of tumors where detection category determined the time of diagnosis irrespective of the underlying carcinogenic process.

The prospective analyses of gene expression levels in the years preceding breast cancer diagnosis as assessed by the log-fold change between cases and controls showed significant differences in the curve groups after stratification by lymph node status and detection category. The analyses showed the ability to discriminate between different stages of the carcinogenic process. A previous analysis of a case-control study within NOWAC showed that differences in gene expression mainly reflect immune responses, but also genes related to cell control [24]. The analyses of trajectories could aid in understanding the time dependent interaction between the immune response and carcinogenesis. Our findings should be further interpreted in relation to the biology of both single genes and gene pathways.

An agnostic search for time trends depends on a sensitive statistical approach. We have presented two novel statistical methods that demonstrated that the gene expression levels varied over time in the last years before breast cancer diagnosis and that the development over time differed by lymph node status among women who attended the National Breast Cancer Screening Program in Norway (i.e., those with screen-detected or interval cancers). One of the methods focused on identifying genes with specific changes over time within a given lymph node status. The other method focused on differences in gene expression levels between lymph node statuses in the different time periods. Both methods focused on different aspects of functional time dependency of gene expression levels relative to time of breast cancer diagnosis, and both methods gave significant results when many genes were used. As gene expression data are very noisy, our methods used information from several genes simultaneously to increase the power of the hypothesis tests.

A potential weakness of the curve group approach is the increasing number of curve groups as observation time periods increases. When there are four time periods, 24 curve groups will be needed, and even more will be needed for five time periods.

Studies of gene expression levels in peripheral blood are challenging and have many difficulties and pitfalls. Most biobanks suffer from ubiquitous degradation by RNase, which reduces the quality of mRNA for whole genome analyses. Only samples that contain a specific buffer or are directly frozen in liquid nitrogen can be used for whole genome analyses. The signals related to carcinogenesis in the blood are expected to be much weaker than those in tumor tissue and can be confounded by signals from exposures to carcinogens or other lifestyle factors. The problem of noise due to the complicated study of carcinogenesis, the need for an adequate epidemiological design including exposure information and blood sampling, complicated technology, and the development of robust statistics, could make the approach unsuccessful. The prospective design of our study made it difficult to increase the statistical power, so our results should be interpreted with care.

To the best of our knowledge, the NOWAC postgenome cohort is the largest population-based prospective cancer study designed for transcriptomics due to the presence of buffered RNA. All parts of the analyses were done within the framework of the NOWAC study. In the NOWAC postgenome cohort, a single laboratory processed all samples using the same technology, thus reducing analytical bias and batch effects. The cohort design reduced selection bias. A weakness of a prospective study could be possible changes in case-control status as controls became cases over time, thus reducing the differences in gene expression levels within a case-control pair. We removed all case-control pairs in which controls were diagnosed with breast cancer or any other cancer within 2 years of blood sampling. The matching was done only for storage time and year of birth. Matching on other variables will eliminate the inclusion of these lifestyle factors in the analyses. If matched on e.g. smoking we could not estimate the effect of smoking or any interactions with other risk factors. Unfortunately, there was no repeated sampling of blood, and no additional questionnaires were completed. Repeated measurements would secure better analyses, making it possible to use intra-individual comparisons over time.

## Conclusions

The proposed statistical methods are sensitive for finding curve groups of genes even for strata with few case-control pairs. This made it possible to describe and test non-linear relationships. Our findings could be viewed as a proof of concept of systems epidemiology, indicating the potential to include gene expression for functional analysis in prospective studies of cancer.

## Abbreviations

NOWAC: Norwegian Women and Cancer
REK: Regional Ethical Committee
